# Exosomal TACSTD2 promotes invasion, metastasis and glycolysis in ovarian cancer

**DOI:** 10.1007/s12672-025-04041-6

**Published:** 2025-12-02

**Authors:** Qisheng Cheng, Xinyuan Zhang, Joseph Adu-Amankwaah, Yanyu Li, Meng Wang, Jingbo Zhang, Qing Wang, Xueyan Zhou, Zeyuan Yin, Bei Zhang

**Affiliations:** 1 Suzhou Medical College of Soochow University, Suzhou, China; 2 Department of Obstetrics and Gynecology, The Affiliated Taizhou People’s Hospital of Nanjing Medical University, Taizhou, China; 3 Jiangsu Key Laboratory of New Drug Research and Clinical Pharmacy, Xuzhou Medical University, Xuzhou, China; 4 Department of Physiology, Xuzhou Medical University, Xuzhou, China; 5 Department of Obstetrics and Gynecology, Xuzhou Central Hospital, Xuzhou Clinical School of Xuzhou Medical University, Xuzhou, China; 6 The First Clinical Medical School, Xuzhou Medical University, Xuzhou, China; 7 Department of Cardiology, The Affiliated Hospital of Xuzhou Medical University, Xuzhou, China

**Keywords:** Ovarian cancer, Exosomes, TACSTD2, Metastasis, Glycolysis

## Abstract

**Background:**

Tumour-derived exosomes are involved in various cancer processes, including invasion, metastasis, and tumour microenvironment (TME) remodelling. However, the function and mechanism of exosomes in ovarian cancer (OC) are still under investigation. The present study investigated the effects of tumour-derived exosomes on the carcinogenesis and progression of OC.

**Methods:**

OC gene expression profiles were obtained from The Cancer Genome Atlas (TCGA) database and two independent Gene Expression Omnibus (GEO) datasets (GSE7463 and GSE12470). The exosome related genes were obtained by intersecting with OC related exosomes in the ExoCarta database, subsequently, the exosome genes of interst were identified through performing survival analysis in the GEPIA database.The expression of genes selected were validated in OC cell lines (SKOV3, OVCAR3 and A2780) and ascites of OC patients through western blot and PCR. And further knockdown and overexpression of this gene were performed in OC cells to detect their effects on cell proliferation, migration, and invasion. Subsequently, the potential biological functions and regulatory mechanisms were explored based on the Gene Set Enrichment Analysis (GSEA), and changes in relevant target proteins were validated through western blot. Furthermore, SKOV3-luc + cells were injected intraperitoneally into female BALB/c nude mice to construct an in orthotopic xenograft mouse model, in order to evaluate the effect of differential genes on in vivo OC.

**Results:**

Based on the results of bioinformatics analysis, TACSTD2 was selected for experimental validation. Cell function experiments verified that exosomal TACSTD2 promoted proliferation and metastasis by mediating glycolysis in OC. Animal experiments indicated that exosomal TACSTD2 promoted abdominal metastasis in OC. The in vitro and in vivo experimental results were consistent with the bioinformatics analysis.

**Conclusion:**

TACSTD2, which can be detected in ovarian cancer-derived exosomes, plays an important role in the invasion, migration and glycolysis in ovarian cancer. Furthermore, we have discovered that TACSTD2 may promote the progression of ovarian cancer by regulating glycolysis. Further research will enable its potentially prognostic marker and therapeutic target for ovarian cancer.

**Supplementary Information:**

The online version contains supplementary material available at 10.1007/s12672-025-04041-6.

## Background

Ovarian cancer (OC) has the highest mortality rate among gynaecological cancers [1]. According to the latest statistical analysis of cancer in the United States, there are estimated to be 19,710 new cases of OC and 13,270 deaths related to OC in 2023. Thus, OC is the fifth leading cause of death among female cancer patients and the most common cause of gynaecological cancers [2]. Due to the absence of early symptoms and effective diagnostic methods, more than 70% of patients are diagnosed with advanced-stage OC (FIGO stages III-IV) [3]. Patients diagnosed with advanced OC often experience a poor prognosis, characterized by extensive peritoneal dissemination, massive ascites, resistance to chemotherapy, and a five-year survival rate of less than 25% [4]. Although new therapies, such as targeted therapy and immunotherapy, have emerged in recent years, the current treatment options for OC remain limited [5]. Therefore, it is crucial to explore specific diagnostic biomarkers and potential therapeutic interventions for OC is particularly important.

Exosomes are membranous vesicles with diameters ranging from 40 to 160 nm (average of 100 nm). Exosomes share the same topology as cells, and they are enriched with lipids, nucleic acids, and protein complexes [6, 7]. Research has demonstrated that tumour cells secrete more exosomes than normal cells, and exosomes derived from tumour cells can promote tumour progression by altering the local and distant microenvironments [8]. Recent studies have provided evidence for the homing properties of tumour-derived exosomes, which is referred to as “tumour-derived exosome tropism” [9–11]. As a result of this tropism, tumour-derived exosomes exhibit a significantly greater degree of targeting towards their parental cancer cells than to nontumor organs [9–12]. This tropism has a profound impact on tumour proliferation, drug resistance, and metastasis.

Studies have shown that exosomes in the tumour microenvironment (TME) often promote OC metastasis [13–15]. In the present study, mRNAs and proteins from OC-derived exosomes were investigated, and the OC sequencing data from The Cancer Genome Atlas (TCGA) database and two independent Gene Expression Omnibus (GEO) datasets were analysed. TACSTD2 was significantly overexpressed in the tumour tissues of OC patients compared to those of healthy controls. This overexpression was also correlated with survival prognosis. Research indicates that TACSTD2 is connected to metastasis and serves as a poor prognosis indicator in OC [16]. It has been discovered that overexpression of TACSTD2 causes resistance to cisplatin, with the mechanism being mediated by the Rap1/PI3K/AKT signaling pathway in high-grade serous ovarian carcinoma (HGSOC) [17]. TACSTD2 has been identified as a HGSOC-specific extracellular vesicles protein marker and the expression of TACSTD2 is specifically increased on extracellular vesicles harvested from HGSOC patient ascites [18]. However, there is limited research on the impact of TACSTD2 on the proliferation, migration, and invasion ability of OC.

The role of glycolysis in tumor progression has received increasing attention in recent years. Our study found that OC-derived exosomal TACSTD2 may influence the proliferation and metastasis of OC by regulating glycolysis or through the ERBB2/PI3K/AKT/FOXO1 signaling pathway. These findings may offer new mechanistic insights for the treatment of ovarian cancer.

## Methods

### Data collection and processing

We obtained mRNAs and proteins from OC-derived exosomes by accessing the ExoCarta database (http://www.exocarta.org/) and retrieved transcriptomic data from the TCGA database (https://portal.gdc.cancer.gov/). Furthermore, we downloaded other OC-related datasets, namely GSE12470 (*n* = 53) and GSE7463 (*n* = 43), from the GEO database (https://www.ncbi.nlm.nih.gov/geo/).

The datasets from GEO and TCGA were standardized by “normalizeBetweenArrays” command in the “limma” package in R (v4.0.3) software, and the datasets were normalized using the “calcNormFactors” function in the “edgeR” package. Furthermore, the “removeBatchEffect” function in the “limma” package was used to eliminate the batch effects of the data. Finally, we screened the differentially expressed genes (DEGs) between OC samples and normal samples using “limma” package. The screening criteria were log2-fold change ≥ 1 and adjusted *P*-value < 0.05. Finally, we identified DEGs associated with exosomes in OC. To explore the associated biological signaling pathways and mechanisms, we performed a genome enrichment analysis (GSEA) of the TACSTD2 high- and low- expression groups using GSEA software (v4.1.0), which mainly relies on the “clusterProfiler” package [19, 20]. We investigated the effect of the top 10 upregulated genes on overall survival in OC patients using GEPIA (http://gepia.cancer-pku.cn/). *P*-values < 0.05 were considered to indicate statistical significance. Finally, we identified the target genes associated with survival.

### Cell culture

OVCAR-3 cell line was purchased from Dalian Bergolin Biotechnology Co., Ltd (Dalian, China). OC cell lines (SKOV3, OVCAR3, A2780) and a normal ovarian cell line (IOSE-80) were provided by Dr. Xueyan Zhou group from the Xuzhou Medical University. All cell lines were cultured in RPMI 1640 medium (Biochannel, Nanjing, China) supplemented with 10% fetal bovine serum (FBS) (CLARK, Virginia, USA). The cells were incubated at 37 °C with 5% CO2.

### Exosome extraction

The OC cells were cultured in a complete medium until they reached 70%-80% confluence (10 cm dish). The culture supernatant was collected and centrifuged at 300 × g for 10 min at 4 °C to remove dead cells and debris. Next, the culture supernatant was centrifuged at 2,000 × g for 10 min at 4 °C to remove biopolymers and apoptotic bodies. The biopolymers and apoptotic bodies were removed from the culture serum by utilizing Millipore sterile filters with a pore size of 0.22 μm (Beyotime, Shanghai, China) to eliminate large vesicles, particles, and bacteria. The filtered supernatant was centrifuged at 10,000 × g for 30 min at 4 °C to eliminate cell debris, large vesicles, and impurities. The sediment at the bottom of the tube was discarded, and the supernatant was subsequently centrifuged at 100,000 × g for 90 min at 4 °C. Then, the supernatant was discarded, and the exosome precipitate was resuspended in 50–100 µl of PBS.

### Dynamic light scattering (DLS) analysis

The extracted exosome precipitate was resuspended in 100 µl of PBS and stored at 4 °C. We used a laser nanoparticle size/potentiometer (380ZLS, PSS) to analyze the size distribution of exosomes. After adjusting the parameters of the instrument, the purified samples were added to analyze the diameter of the exosomes.

### Transmission electron microscopy (TEM)

Transmission electron microscopy (TEM, FEI Teneo VS, Hillsboro, USA) was used to identify the form and structure of the exosomes. A copper mesh, covered with carbon to match the electron microscope, was filled with approximately 10–20 µl of exosome solution. The liquid from the filter was removed using filter paper. Subsequently, the exosomes were negatively stained for 20 min using a 3% phosphotungstic acid dye solution (20–50 µl) and then washed three times with PBS after the filter paper had dried. The exosomes were observed using a TEM after drying at room temperature.

### Exosome fluorescence labelling uptake experiment

First, exosome samples were labelled with a red fluorescent probe for cell membranes (Dil, Red, Beyotime, Shanghai, China) and incubated at 37 °C for 20 min. Following incubation, the labelled exosomes were purified by ultracentrifugation for 70 min at 110,000 × g at 4 °C. The exosome pellet was resuspended in 1 × PBS. Subsequently, SKOV3 and OVCAR3 cells were incubated separately with Dil-labelled purified exosomes for 24 h at 37 °C. Following the 24-hour incubation, the cells were washed three times with 1× PBS for 3 min each. Next, the cells were fixed with 4% paraformaldehyde for 10 min and washed three times with 1× PBS for 3 min each. Finally, the cells were stained with DAPI for 5 min and washed three times with 1× PBS for 3 min each. Finally, the cells were observed for exosome uptake using a fluorescence microscope (Olympus BX43 + DP74, Tokyo, Japan).

### Lentiviral production and infection

To stably transfect SKOV3 and OVCAR3 cells, a lentivirus-mediated vector containing the TACSTD2 gene, designed to either inhibit or augment its expression, was obtained from GeneChem Co., Ltd (Shanghai, China). The transfections were conducted following the manufacturer’s instructions. In brief, the lentivirus volume for transfection was calculated based on the infection values of the cells, the cell number at the time of transfection, and the lentivirus titre. After 48 h of transfection, the stably transfected cells were screened with puromycin (3 µg/ml) and confirmed by Western blot and real-time PCR.

### RNA extraction and RT-PCR analysis

Total RNA was extracted using RNA-easy Isolation Reagent (Vazyme, Nanjing, China) and quantified. Reverse transcription was conducted according to the instructions of the SweScript RT I First Strand cDNA Synthesis Kit (Servicebio, Wuhan, China). The following procedure was implemented using 2 × SYBR Green qPCR Master Mix (Servicebio, Wuhan, China). PCR was carried out using a fluorescence quantitative PCR instrument (QuantStudio™ 3 System). The data were statistically analysed with GAPDH as the internal control and 2^−ΔΔCt^ as the method for calculating relative expression. The sequences of the primers used were as follows: GAPDH forward primer: 5′-TTCGACAGTCAGCCGCATCTTCTT-3′; GAPDH reverse primer: 3′-CAGGCGCCCAATACGACCAAATC-5′; TACSTD2 forward primer: 5′-GAATCCATTGCGACATTGTGAAGGC-3′; and TACSTD2 reverse primer: 5′-ACAAACTCCTCTTCTCCTCGGGTAG-3′. The sequences used for TACSTD2-targeting short hairpin RNA (shRNA) were as follows: shRNA#1: CTCCAAGTGTCTGCTGCTCAA; shRNA#2: GAGCGCACGCTCATCTATTAC; shRNA#3: CGCCTTCAACCACTCAGACCT; and shRNA-NC: TTCTCCGAACGTGTCACGT.

### Western blot analysis

The cell pellet was lysed using RIPA lysis buffer supplemented with phenylmethylsulfonyl fluoride (PMSF) at 4 °C for 20 min. Following centrifugation at 4 °C and 12,000 × g for 5 min, the protein concentration in the supernatant was determined using a bicinchoninic acid assay (BCA; Beyotime, Shanghai, China). The proteins were resolved on a 10% SDS-PAGE gel and subsequently transferred onto a PVDF membrane with a pore size of 0.45 μm (Beyotime, Shanghai, China). Following blocking with 10% blocking buffer (Beyotime, Shanghai, China), the membranes were incubated with specific antibodies overnight at 4 °C. The immunoblots were probed with the following primary antibodies: TACSTD2 (1:1000; Bioworld, Minisuda, USA), GAPDH (1:10000; Bioworld, Minisuda, USA), CD9 (1:1000; Proteintech, Wuhan, China), CD63 (1:200; Proteintech, Wuhan, China), TSG101 (1:2000; Proteintech, Wuhan, China), ERBB2 (1:2000; Proteintech, Wuhan, China), PI3K (1:1000; Abmart, Shanghai, China), p-PI3K (1:500; Affinity, Cincinnati, USA), AKT (1:1000; Abmart, Shanghai, China), p-AKT (1:1000; Abmart, Cincinnati, USA), FOXO-1 (1:1000; Affinity, Cincinnati, USA), p-FOXO-1 (1:1000; Affinity, Cincinnati, USA), GLUT1 (1:1000; Affinity, Cincinnati, USA), HK2 (1:1000; Affinity, Cincinnati, USA), PKM2 (1:1000; Affinity, Cincinnati, USA), and LDHA (1:1000; Affinity, Cincinnati, USA). After the PVDF membranes were washed three times with 1× Tris-buffered saline and Tween 20 for 5 min, they were incubated with HRP-conjugated AffiniPure goat anti-rabbit IgG(H + L) secondary antibody (1:10000, Proteintech, Wuhan, China) for 1 h at room temperature. After three washes with 1× Tris-buffered saline (TBS) and Tween 20 for 5 min each, the protein bands were visualized using ECL Western Blotting Substrate (Affinity, Cincinnati, USA).

### Cell counting Kit-8 (CCK-8) assay

Cells in the logarithmic growth phase were trypsinized and resuspended to obtain a single-cell suspension. Each well of a 96-well plate was seeded with 1000 cells/100 µl, and added serum-free medium containing 50 µg/ml of exosomes. The 96-well plate was incubated in a cell incubator at 37 °C for 4 h, and the time immediately after adherence (0 h) was noted. Each well was supplemented with 10 µl of CCK-8 reagent (Meilunbio, Dalian, China) solution, and the cells were further incubated at 37 °C for 2 h. The absorbance of the cells was measured at an optical density of 450 nm after 24, 48, and 72 h.

### Detection of lactate production, ATP production, and glucose uptake

OC cells were cultured in 6-well plates until they were 90% confluent, then cultured for 48 h at 37 °C in a serum-free medium, which containing exosomes (50 µg) in each chamber. Lactate production, ATP synthesis, and glucose consumption in OC cells were measured using a lactate assay kit, ATP assay kit, and glucose assay kit (JianCheng, Nanjing, China), respectively, based on the manufacturer’s instructions.

### Wound-healing assay

After being digested with trypsin, log-phase cells were seeded into six-well culture plates. When the cells reached 90% confluence, the cell layer was gently scraped using a 100 µl micropipette tip. Floating cells were subsequently rinsed with PBS, and the cell layer was then cultured for 48 h at 37 °C in a serum-free medium, which containing exosomes (50 µg) in each chamber. The ability of the cells to heal wounds was observed and recorded using a microscope and camera at both 0 and 48 h.

### Transwell assay

The invasion ability of the cells was assessed by Transwell assay in a 24-well culture plate. Matrigel (Corning Coster) was diluted with a serum-free medium at an 8:1 ratio. The cells in the exponential growth phase were regularly digested, and the cell concentration was adjusted to approximately 10^5^ cells/ml. Approximately 200 µl (approximately 2 × 10^4^ cells) of cell suspension was added to the upper layer of the Transwell chamber. Each upper chamber was supplemented with serum-free medium containing exosomes (50 µg). In the lower chamber, 500 µl of medium supplemented with 10% FBS was added. Following incubation at 37 °C for 48 h, the cells in the Transwell chamber were fixed with 4% paraformaldehyde for 20 min and subsequently stained with crystal violet for 30 min. Using a cotton swab, the Matrigel and cells from the upper surface of the chamber were removed. The stained cells were observed and enumerated using a microscope.

### Mouse abdominal implant tumor model

All experiments followed the institutional guidelines and regulations, and the authors complied with the ARRIVE guidelines. Female BALB/c nude mice, aged four to six weeks, were obtained from Zhejiang Vital River Laboratory Animal Technology Co., Ltd. The mice used to establish the abdominal implant tumor model of OC were bred under specific pathogen-free (SPF) conditions. The SKOV3 cell line was transduced with a lentiviral luciferase reporter gene, screened with puromycin, and selected to obtain monoclonal cells referred to as SKOV3-luc + cells. SKOV3-luc + cells in the logarithmic growth phase were trypsinized, suspended in a serum-free medium, and utilized at a density of 1 × 10^8^ cells/ml. Each nude mouse was intraperitoneally injected with 100 µl of cell suspension. An orthotopic xenograft mouse model and live bioluminescence imaging were employed to assess the impact of exosomal TACSTD2 on OC in vivo. Ten nude mice were randomly assigned to two groups, with five mice in each group. 50 µg of exosomes was used based on literature [21–23], with Gupta et al. (2021) reporting this dosage as safe and effective for intraperitoneal injection in mice, eliciting measurable biological effects [21]. One group received intraperitoneal injections of 50 µg of exosomes derived from the SKOV3-TACSTD2-OE group, while the control group received intraperitoneal injections of 50 µg of exosomes from the SKOV3-vector group every 3 days. The animals were cared for according to laboratory protocols. Live bioluminescence imaging was conducted at the end of 8 weeks. The nude mice were anaesthetized with 2% isoflurane and 1.5 L/minute of oxygen and then intraperitoneally injected with a 150 mg/kg dose of D-luciferin. Ten minutes after substrate injection, the nude mice were positioned prone in the imaging chamber. An in vivo imaging bio-instrument was used to detect the intensity and location of the luminescence. The animals were euthanized as necessary, and the tumors were dissected, weighed, and photographed for documentation purposes. Mice were ethically euthanized through carbon dioxide inhalation. We observed the animals for dilated pupils and response to stimuli. We ensured a thorough determination of the cessation of vital signs and confirmed the humane and ethically sound completion of the euthanasia procedure.

### Statistical analysis

The data are presented as the mean values ± SDs and were analysed for significance using an unpaired t-test conducted with GraphPad Prism 6 software (GraphPad Software Inc., CA). *P* < 0.05 was considered representative of a significant difference. The correlation coefficient was analysed using Pearson’s test, and a two-tailed p- value less than 0.05 was considered to indicate statistical significance.

## Results

### Identification of Exosomal DEGs in OC

**Fig. 1:**
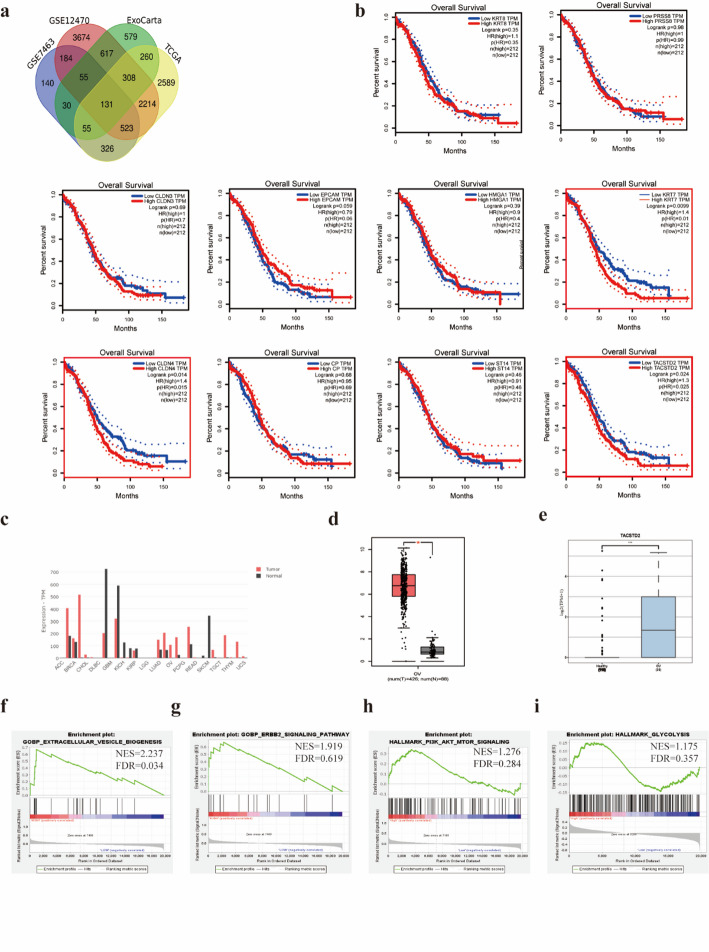
Identification of exosomal DEGs in OC and analysis of the biological role of TACSTD2 in OC. **a** 131 exosome-associated DEGs in ovarian cancer. **b** Kaplan–Meier analysis for OS of OC patients based on the mRNA expression of the top 10 upregulated genes. **c** The expression of TACSTD2 in pan-cancer based on the GEPIA database. **d** The expression of TACSTD2 in OC based on the GEPIA database. **e** The expression of TACSTD2 in ovarian cancer-derived exosomes based on the exoRBase 2.0 database. **f**–**i** GSEA enrichment based on TCGA-OC samples

The DEGs in OC samples and normal ovarian tissue samples were evaluated using the GEO (GSE7463 and GSE12470 datasets) and TCGA databasets. The screening conditions were set as |log2FC| ≥ 1 and adjusted *P*-value < 0.05, and the data were analysed using R v4.0.3 software. The obtained DEGs were intersected with genes in OC-derived exosomes collected from the ExoCarta database, which yielded 131 exosome-associated DEGs (Fig. 1A) (Supplementary material 2). To identify key genes associated with the prognosis of OC patients, survival analysis of the top 10 upregulated differentially expressed exosomal genes in OC was performed using GEPIA **(**Supplementary material 1). There was a statistically significant positive correlation of only CLDN4, KRT7, and TACSTD2 with the survival of OC patients (Fig. 1B). Survival curves indicated that high expression of these three genes was associated with shorter overall survival (OS) and predicted a poor prognosis. Based on literature and functional predictions of these genes, which indicate a close association between TACSTD2 and poor prognosis in OC [24–27]. We focused on studying TACSTD2.

Based on the literature and functional prediction of these genes, the present study focused on TACSTD2. According to the GEPIA database, TACSTD2 was highly expressed in various solid tumours, including OC, breast cancer, and bladder cancer (Fig. 1C). To further investigate the significance of TACSTD2 in OC, the expression of TACSTD2 was analysed in 426 °C samples and 88 normal ovarian tissue using the GEPIA database. TACSTD2 was significantly upregulated in OC tissues compared to normal ovarian tissues (Fig. 1D, *p* < 0.05). The exoRBase 2.0 database was utilized to explore the expression of TACSTD2 in OC-derived exosomes. Exosomal TACSTD2 was expressed at significantly greater levels in OC-derived exosomes than in normal cellular exosomes (Fig. 1E, *p* < 0.05).

### Analysis of the biological role of TACSTD2 in OC

**Fig. 2:**
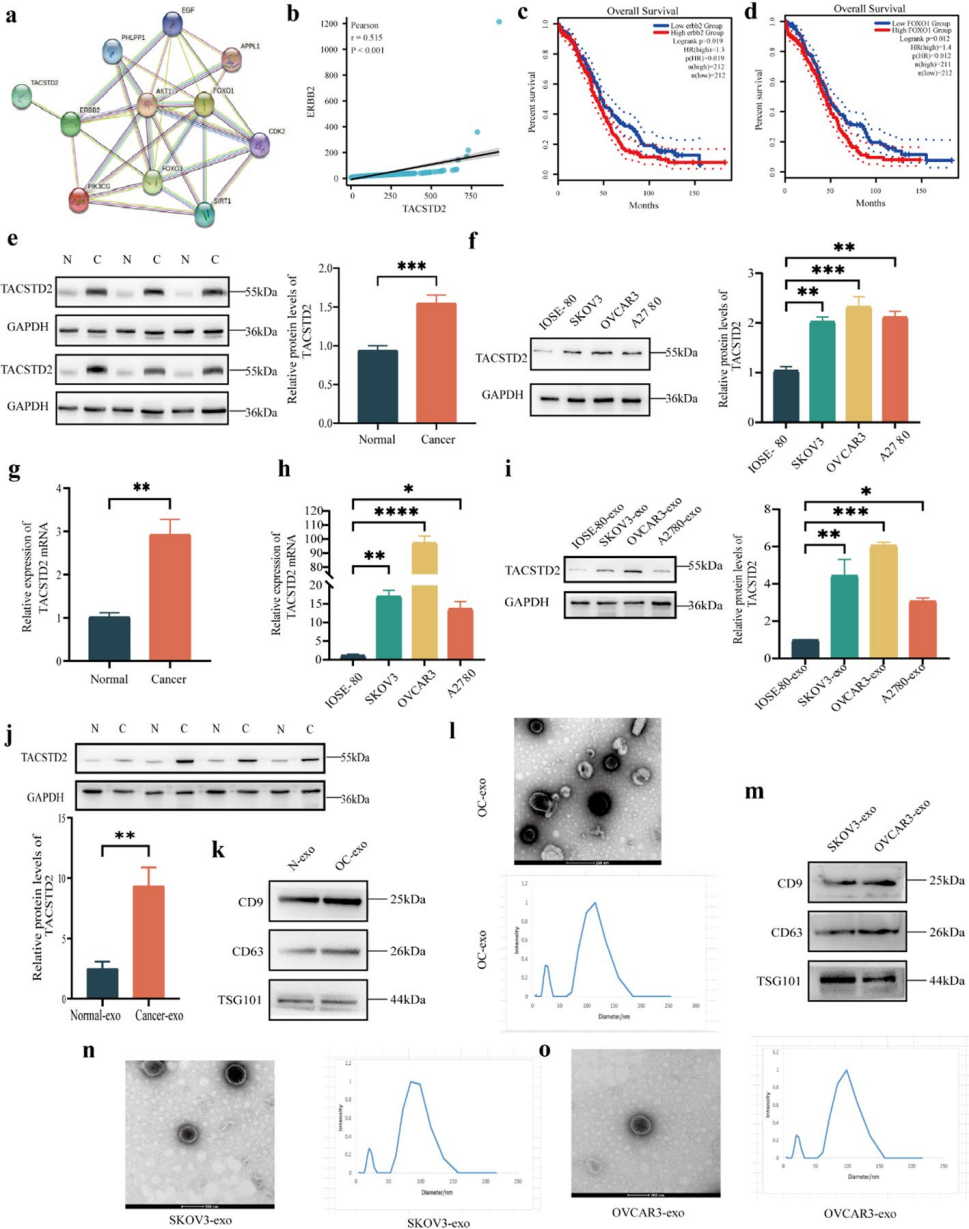
The potential protein interactions of TACSTD2 and the expression of TACSTD2 in OC tissues and cells. **a** The potential protein interactions of TACSTD2 through the STRING. **b** The correlation analysis of TACSTD2 and ERBB2 based on TCGA. **c**,** d** Kaplan–Meier analysis of OS by ERBB2 and FOXO1 expression in OC. **e**,** f** The expression of TACSTD2 in OC tissues (*n* = 6) and OC cells detected by Western blot. **g**,** h** The expression of TACSTD2 in OC tissues and OC cells detected by real-time PCR. **i**,** j** The expression of exosomal TACSTD2 in OC cells and OC ascites(*n* = 4) detected by Western blot. **k** Expression of exosome molecular marker proteins CD9, CD63, and TSG101 in the exosomes derived from normal ascites and OC ascites detected by Western blot. **l** Exosomes derived from OC ascites were characterized by transmission electron microscope (TEM) and dynamic light scattering (DLS) analysis. **m** Expression of exosome molecular marker proteins CD9, CD63, and TSG101 in the exosomes derived from SKOV3 and OVCAR3 cells detected by Western blot. **n**,** o** Exosomes derived from SKOV3 and OVCAR3 cells were characterized by transmission electron microscope (TEM) and dynamic light scattering (DLS) analysis. *p < 0.05, **p < 0.01, ***p < 0.001

To predict the role and mechanism of TACSTD2 in OC development, GSEA was conducted on OC samples from high and low TACSTD2 expression groups in the TCGA database. High TACSTD2 expression was associated with extracellular vesiculogenesis bioprocesses (Fig. 1F, *p* < 0.05), including exosomes, apoptotic vesicles, and microvesicles. Additionally, GSEA suggested the potential pattern that high TACSTD2 expression was linked to ERBB2-mediated cellular signaling pathways (Fig. 1G) and PI3K/AKT-regulated signaling pathways (Fig. 1H). Furthermore, a trend was observed : high expression of TACSTD2 was enriched in the glycolysis pathway (Fig. 1I). To explore potential protein regulatory relationships, the STRING [24] database was used to predict protein interactions (Fig. 2A). TACSTD2, through ERBB2, may further regulate PI3K/AKT pathway-related proteins, thus exerting biological effects. Notably, the PI3K/AKT pathway is a well-established signaling pathway that plays a critical role in the proliferation, metabolism, migration, and invasion of tumour cells.

For additional validation, the expression data of TACSTD2 and ERBB2 in the TCGA OC database were examined, which revealed a significant positive correlation (Fig. 2B, *p* < 0.05). FOXO1, a downstream target gene of AKT, plays a crucial role in regulating various aspects of tumour cell proliferation, the cell cycle, DNA damage repair, and glucose metabolism [25]. The regulation of the PI3K/AKT/FOXO1 signaling pathway has been demonstrated in different types of tumours, including colorectal cancer [26], pancreatic cancer [27], and bladder cancer [28]. Furthermore, analysis of the GEPIA database indicated that high expression of ERBB2 and FOXO1 was associated with shorter OS and predicted a poor prognosis in patients with OC (Fig. 2C, D, *p* < 0.05).

### Expression of Exosomal TACSTD2 in OC

To investigate the role of TACSTD2 in OC and its regulatory mechanism, Western blot, and real-time PCR analyses revealed that TACSTD2 expression was significantly greater in OC tissues than in normal ovarian tissues at both the protein and mRNA levels (Fig. 2E, G, *p* < 0.05). For in vitro validation, the expression of TACSTD2 was evaluated in normal ovarian epithelial cells (IOSE-80) and human OC cells (SKOV3, OVCAR3, and A2780) by Western blot and real-time PCR analyses. TACSTD2 expression was significantly greater in OC cells than in normal ovarian cells at both the protein and mRNA levels (Fig. 2F, H, *p* < 0.05), and the highest TACSTD2 expression was observed in SKOV3 and OVCAR3 cells.

Exosomes were isolated from the cell culture supernatants of IOSE-80, SKOV3, OVCAR3, and A2780 cells using ultracentrifugation. Western blot analysis demonstrated that the levels of the OC-derived exosomal TACSTD2 were significantly greater than the levels of exosomal TACSTD2 protein derived from normal ovarian cells (Fig. 2I, *p* < 0.05). The higher exosomal TACSTD2 expression was observed in OC ascites than in normal ascites (Fig. 2J, *p* < 0.05). Western blot analysis confirmed the presence of exosome-specific protein markers, including CD9, CD63, and tumor susceptibility gene 101 (TSG101), in ovarian cancer (OC) ascites (Fig. 2K). The morphology of the purified exosomes from OC ascites was examined using transmission electron microscopy (TEM), which revealed round or oval microvesicles ranging from approximately 30 to 150 nm in diameter. These microvesicles exhibited teardrop-like or concave hemispherical shapes, characteristic of exosomes (Fig. 2L). Since exosomal TACSTD2 levels were highest in exosomes derived from SKOV3 and OVCAR3 cells, these cell lines were selected for subsequent experiments. Exosomes from both SKOV3 and OVCAR3 cells were characterized in the same manner (Fig. 2M–O). We carefully selected SKOV3 and OVCAR3 cells as ovarian cancer models also based on well-established studies by Hernandez L et al. [29] and Mitra AK et al. [30], which demonstrated that these cell lines, when inoculated subcutaneously in nude mice, exhibit a high tumorigenic rate and express HGSOC markers in transplanted tumor tissue.

### Construction of OC cells stably expressing exosomal TACSTD2

**Fig. 3:**
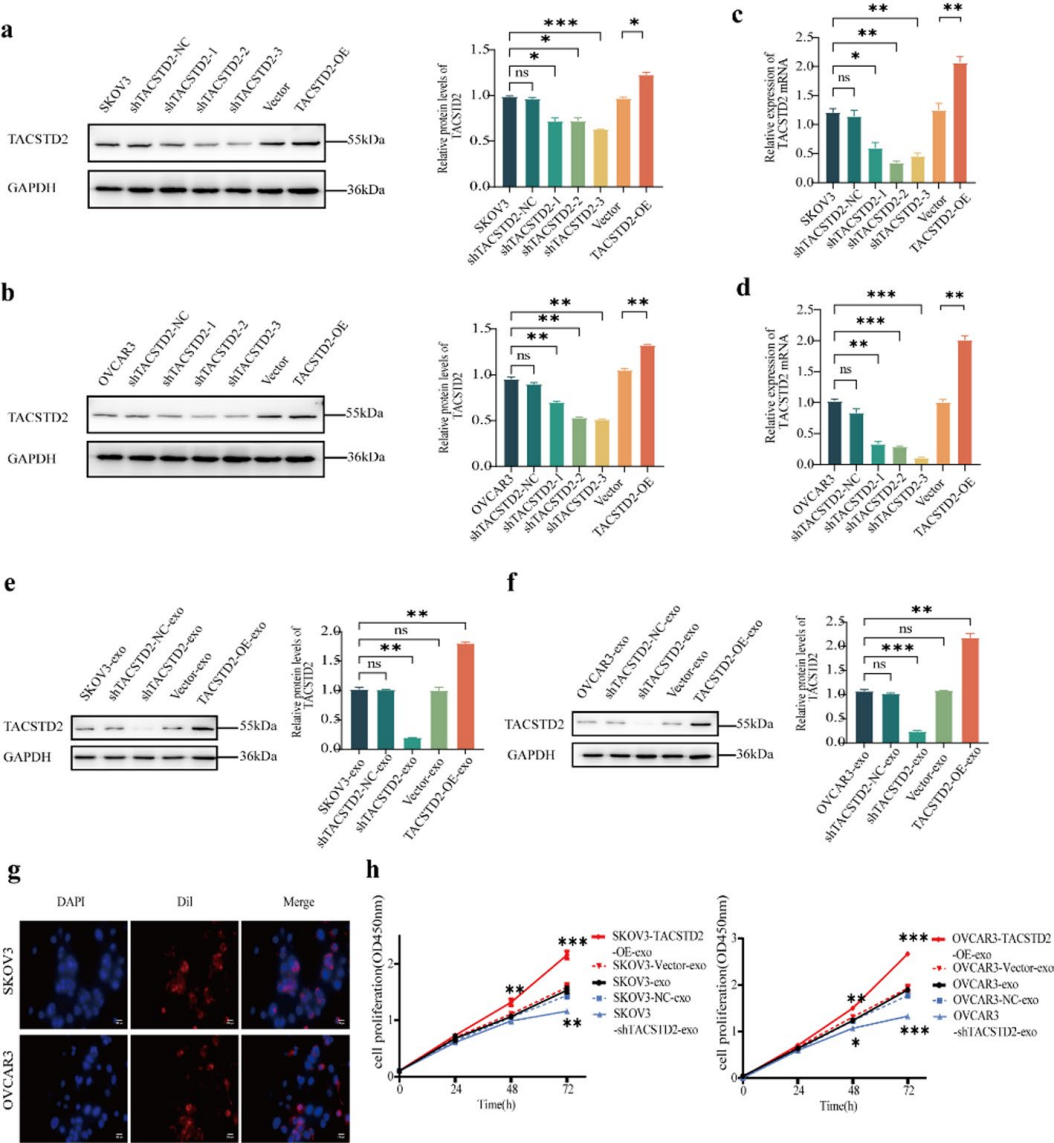
The expression of exosomal TACSTD2 in the treatment designed to OC cells and exosomal TACSTD2 promotes the proliferation of OC cells. **a**,** b** The protein levels of TACSTD2 in SKOV3 and OVCAR3 cells with up- and downregulated TACSTD2 detected by Western blot. **c**,** d** The mRNA levels of TACSTD2 in SKOV3 and OVCAR3 cells with up- and downregulated TACSTD2 detected by real-time PCR. **e**,** f** Changes in exosomal TACSTD2 derived from SKOV3 and OVCAR3 cells with up- and downregulated TACSTD2 detected by Western blot. **g** Exosomes derived from ovarian cancer cells can taken up by OC cells. **h** Effect of exosomal TACSTD2 on the proliferation of OC cells detected by CCK-8 assay. *p < 0.05, **p < 0.01, ***p < 0.001

Lentiviral transduction was used to generate SKOV3 and OVCAR3 OC cell lines with stable overexpression and knockdown of TACSTD2. The transduction efficiency was evaluated using fluorescence microscopy and further validated through Western blot and real-time PCR experiments. Both the protein and mRNA levels of TACSTD2 were significantly lower in SKOV3 and OVCAR3 cells transduced with three different lentiviruses containing knockdown sequences compared to the blank control group (SKOV3 group and OVCAR3 group) and the negative control group (SKOV3-shNC group and OVCAR3-shNC group) (Fig. 3A–D, all *p* < 0.05). The SKOV3-shTACSTD2 group and the OVCAR3-shTACSTD2 group, which exhibited the most significant differences in expression, were selected for subsequent experiments. The protein and mRNA levels of TACSTD2 in the overexpression groups (SKOV3-TACSTD2-OE group and OVCAR3-TACSTD2-OE group) were significantly greater than those in the blank control groups (SKOV3 and OVCAR3 groups) and the vector control groups (SKOV3 vector and OVCAR3 vector groups) (Fig. 3A–D, all *p* < 0.05).

Exosomes from OC cells with either up- or downregulated TACSTD2 expression were subsequently isolated via ultracentrifugation. Exosomal TACSTD2 protein levels were significantly lower in the SKOV3-shTACSTD2-exo and OVCAR3-shTACSTD2-exo groups than in the SKOV3-exo, SKOV3-shNC-exo, OVCAR3-exo, and OVCAR3-shNC-exo groups (Fig. 3E, F, *p* < 0.05). Conversely, the exosomal TACSTD2 protein levels in the SKOV3-TACSTD2-OE-exo and OVCAR3-TACSTD2-OE-exo groups were significantly greater than those in the SKOV3-vector-exo and OVCAR3-vector-exo groups (Fig. 3E, F, *p* < 0.05).

### Exosomal TACSTD2 promotes the proliferation of OC cells

To investigate the uptake of OC-derived exosomes by OC cells, Dil fluorescence-labelled exosomes from OC cells were cocultured with SKOV3 and OVCAR3 cells for 24 h. The exosomes, which were labelled with red fluorescent particles, localized around the nuclei of the blue-labelled OC cells, suggesting the internalization of exosomes and their subsequent functional roles (Fig. 3G).

Following the coculture of up- or downregulated exosomal TACSTD2 with OC cells, a CCK-8 assay was used to assess the impact of exosomal TACSTD2 protein on the proliferative capacity of OC cells. The proliferative ability of OC cells in the SKOV3-shTACSTD2-exo and OVCAR3-shTACSTD2-exo groups was significantly lower than that in the SKOV3-exo, SKOV3-shNC-exo, OVCAR3-exo, and OVCAR3-shNC-exo groups (Fig. 3H, all *p* < 0.05). Conversely, the proliferative ability of OC cells was significantly greater in the SKOV3-TACSTD2-OE-exo and OVCAR3-TACSTD2-OE-exo groups than in the SKOV3-vector-exo and OVCAR3-vector-exo groups (Fig. 3H, *p* < 0.05).

### Exosomal TACSTD2 enhances the migration and invasion of OC cells

**Fig. 4:**
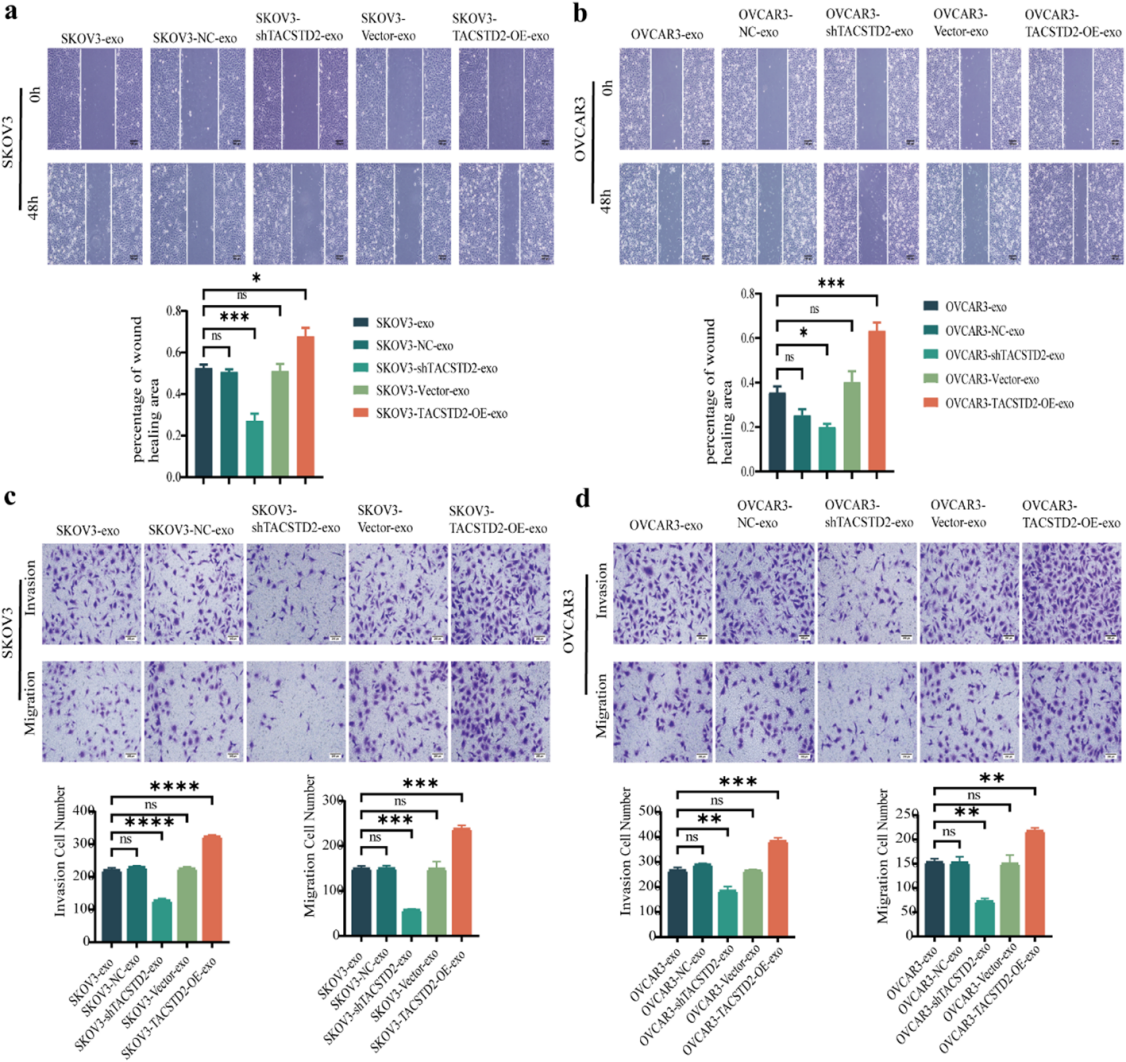
Exosomal TACSTD2 promotes the migration and invasion of OC cells. **a**,** b** Effect of exosomal TACSTD2 on the migration of OC cells detected by scratch assay. **c**,** d** Effect of exosomal TACSTD2 on the migration and invasion of OC cells detected by Transwell assay. *p < 0.05, **p < 0.01, ***p < 0.001

The impact of exosomal TACSTD2 on the biological characteristics of OC cells was further evaluated through co-culture experiments using exosomes derived from OC cells with either up- or downregulated TACSTD2 expression. The scratch assay demonstrated a significant reduction in the migratory ability of OC cells cocultured with SKOV3-shTACSTD2-exo or OVCAR3-shTACSTD2-exo compared to those cocultured with SKOV3-exo, SKOV3-shNC-exo, OVCAR3-exo, or OVCAR3-shNC-exo (Fig. 4A, B, *p* < 0.05). Conversely, the migratory ability of OC cells cocultured with SKOV3-TACSTD2-OE-exo or OVCAR3-TACSTD2-OE-exo was significantly greater than that of SKOV3-vector-exo or OVCAR3-vector-exo-treated cells (Fig. 4A, B, *p* < 0.05).

Additionally, Transwell assays revealed that the migration and invasion abilities of OC cells cocultured with SKOV3-shTACSTD2-exo and OVCAR3-shTACSTD2-exo were significantly lower than those of cells cocultured with SKOV3-exo, SKOV3-shNC-exo, OVCAR3-exo, or OVCAR3-shNC-exo (Fig. 4C, D, all *p* < 0.05). Conversely, the migratory and invasive capacities of OC cells cocultured with SKOV3-TACSTD2-OE-exo and OVCAR3-TACSTD2-OE-exo were significantly greater than those of cells cocultured with SKOV3-vector-exo and OVCAR3-vector-exo (Fig. 4C, D, all *p* < 0.05).

### Exosomal TACSTD2 improves glycolysis in OC cells

Given the characteristic metabolic dysregulation observed in cancer, cancer cells rely heavily on glycolysis to enhance energy production. The increased rate of aerobic glycolysis in tumour cells primarily results from the elevated expression of glycolysis-associated enzymes or transporter proteins [31]. Notably, hexokinase 2(HK2) and pyruvate kinase M2(PKM2) act as the rate-limiting enzymes of glycolysis, while glucose transporter protein type 1(GLUT1) facilitates the entry of extracellular glucose into the cell. Additionally, lactate dehydrogenase A(LDHA) converts pyruvate to lactate. To investigate the influence of exosomal TACSTD2 on glycolysis in OC cells, glucose uptake, ATP production, and lactate production were evaluated by coculturing OC cells with exosomes derived from OC cells with either up- or downregulated TACSTD2 expression.

**Fig. 5:**
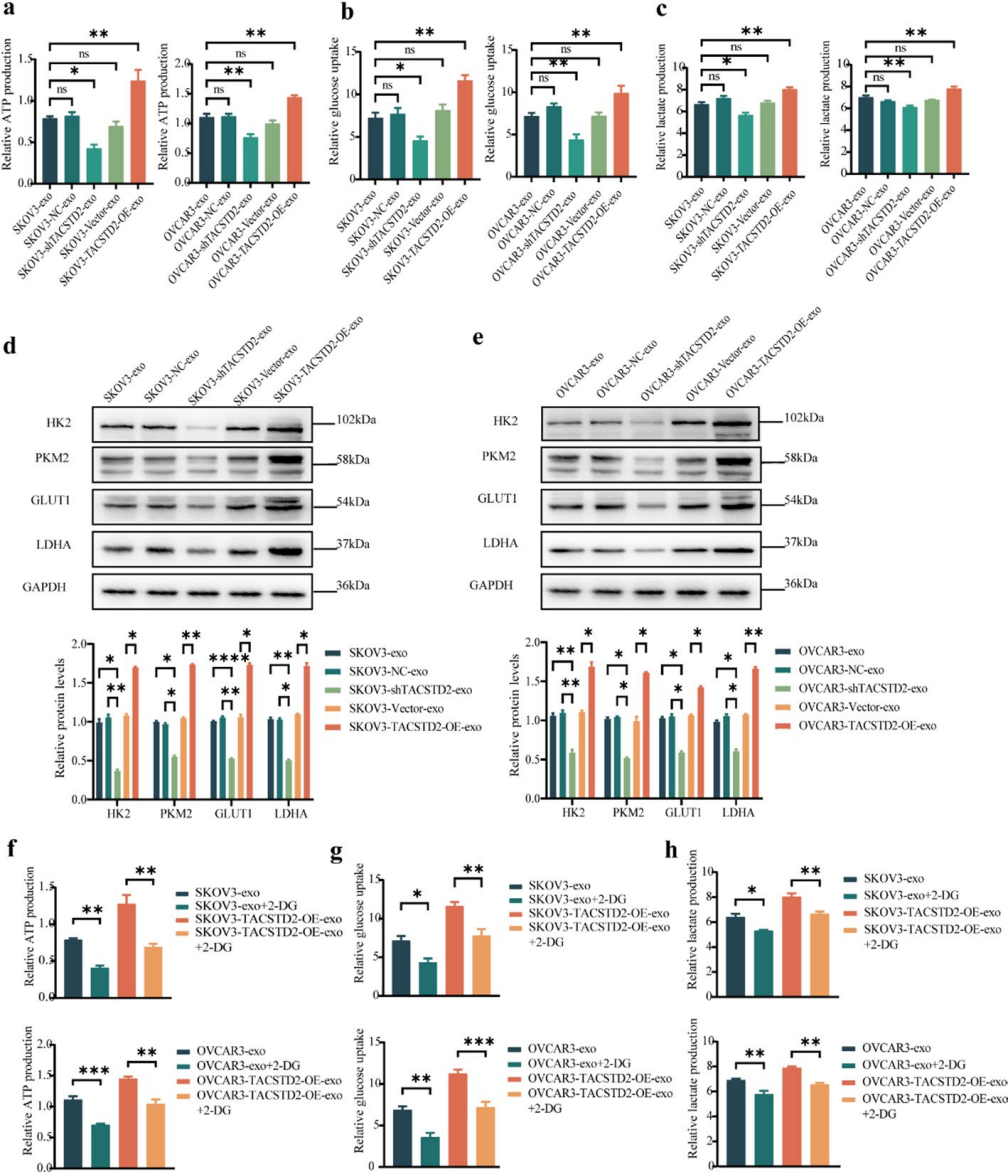
Exosomal TACSTD2 improves glycolysis in OC cells. **a-c** Exosomal TACSTD2 increased ATP production, glucose uptake, and lactate production in OC cells. **d**,** e** The effect of exosomal TACSTD2 on HK2, PKM2, GLUT1, LDHA detected by Western blot. **f-h** The glucose inhibitor 2-DG inhibited ATP production, glucose uptake and lactate production. *p < 0.05, **p < 0.01, ***p < 0.001

Coculture of OC cells in the SKOV3-shTACSTD2-exo and OVCAR3-shTACSTD2-exo groups exhibited significantly lower glucose uptake, ATP production, and lactate production compared to those in the SKOV3-exo, SKOV3-shNC-exo, OVCAR3-exo, and OVCAR3-shNC-exo groups (Fig. 5A–C, all *p* < 0.05). Conversely, compared with those in the SKOV3-vector-exo and OVCAR3-vector-exo groups, the OC cells in the SKOV3-TACSTD2-OE-exo and OVCAR3-TACSTD2-OE-exo groups exhibited significantly increased glucose uptake, ATP production, and lactate production (Fig. 5A–C, all *p* < 0.05). Furthermore, the expression levels of the glycolysis-related proteins, namely, HK2, PKM2, GLUT1, and LDHA, were examined in each group by Western blot analysis. The expression of these four proteins was significantly lower in the SKOV3-shTACSTD2-exo and OVCAR3-shTACSTD2-exo groups than in the SKOV3-exo, SKOV3-shNC-exo, OVCAR3-exo, and OVCAR3-shNC-exo groups (Fig. 5D, E, *p* < 0.05). Conversely, the expression of these proteins was significantly greater in the SKOV3-TACSTD2-OE-exo and OVCAR3-TACSTD2-OE-exo groups than in the SKOV3-vector-exo and OVCAR3-vector-exo groups (Fig. 5D, E, *p* < 0.05).

**Fig. 6:**
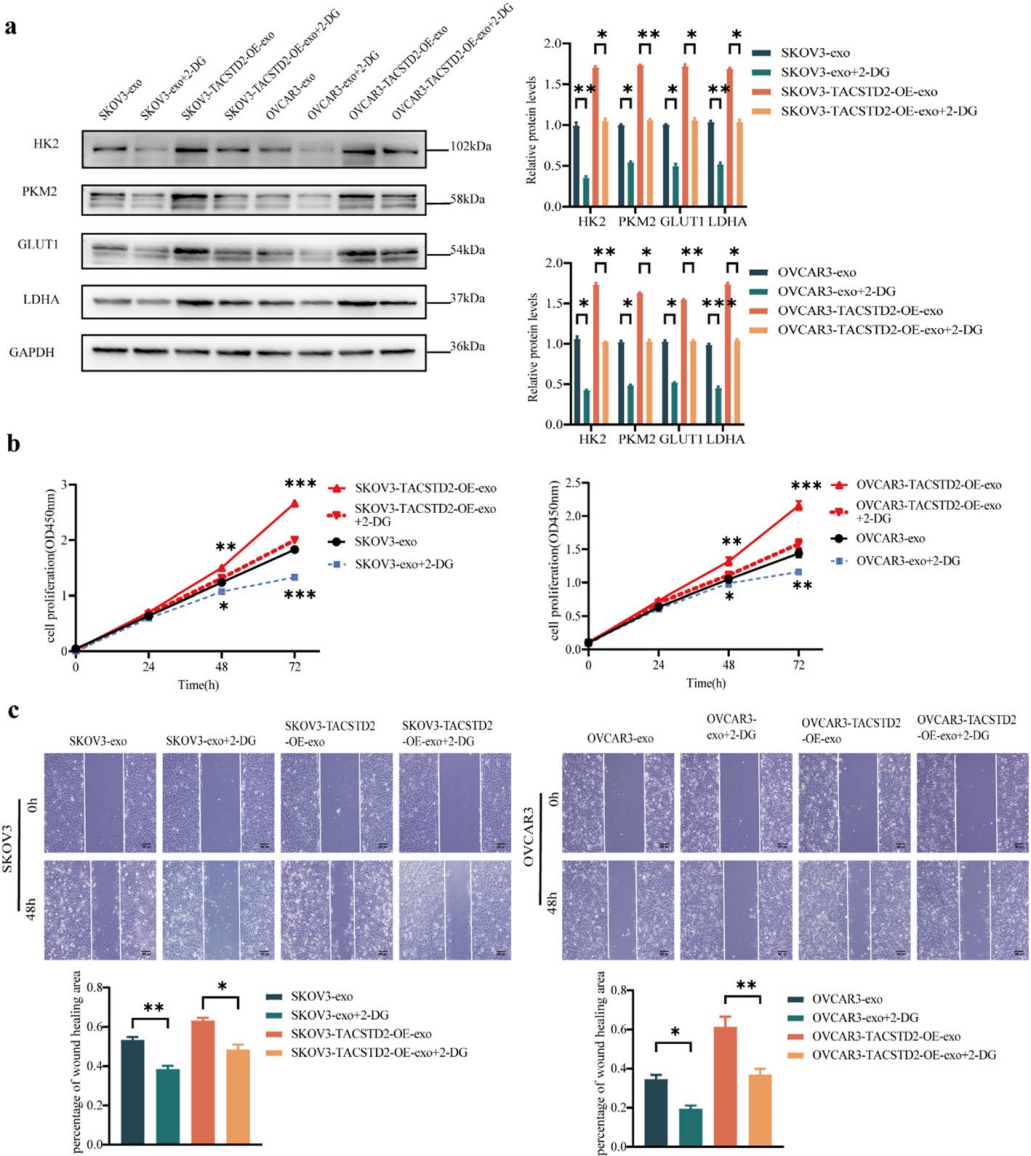
The glucose inhibitor 2-deoxy-D-glucose (2-DG) inhibits glycolysis process and exosomal TACSTD2 promotes progression by mediating glycolysis in OC cells. **a** The effect of 2-DG on HK2, PKM2, GLUT1, LDHA detected by Western blot. **b** The glycolysis inhibitor 2-DG reversed the promoting effect of exosomal TACSTD2 on the proliferation of OC cells detected by CCK-8 assay. **c** The glycolysis inhibitor 2-DG reversed the promoting effect of exosomal TACSTD2 on the migration of OC cells detected by scratch assay. *p < 0.05, **p < 0.01, ***p < 0.001

### Exosomal TACSTD2 promotes progression by mediating glycolysis in OC cells

**Fig. 7:**
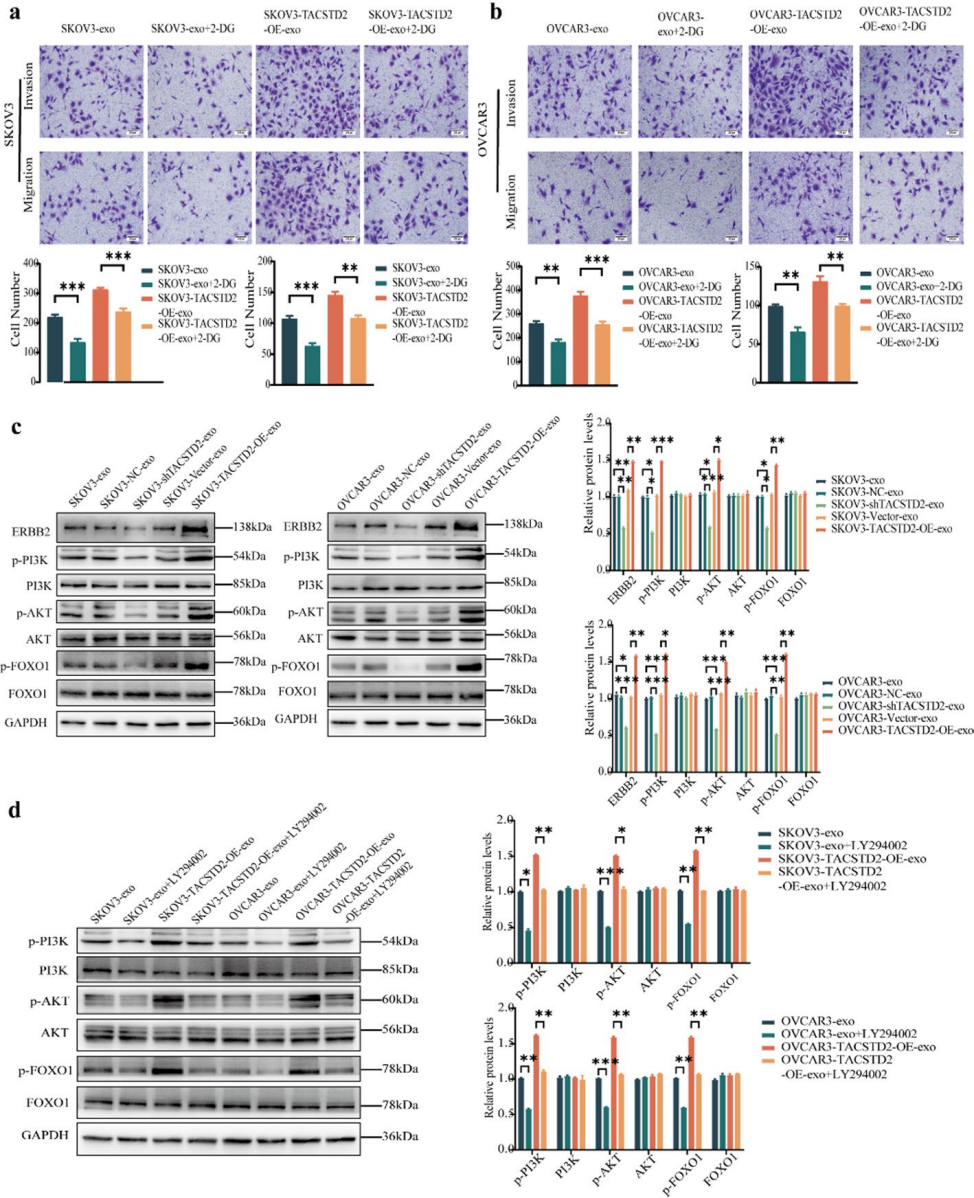
Exosomal TACSTD2 promotes progression by mediating glycolysis in OC cells and exosomal TACSTD2 promotes progression through the ERBB2/PI3K/AKT/FOXO1 signaling pathway in OC cells. **a**,** b** The glycolysis inhibitor 2-DG reversed the promoting effect of of exosomal TACSTD2 on the migration and invasion of OC cells detected by Transwell assay. **c** Effect of exosomal TACSTD2 on ERBB2/PI3K/AKT/FOXO1 signaling pathways in OC cells detected by Western blot. **d** The PI3K inhibitor (LY294002) reversed the overexpression of the pathway proteins p-PI3K, p-AKT, and p-FOXO1 in OC cells detected by Western blot. *p < 0.05, **p < 0.01, ***p < 0.001

To investigate whether exosomal TACSTD2 regulates OC progression through the glycolytic pathway, the present study utilized the 2-deoxy-D-glucose (2-DG) glucose inhibitor. Treatment with 2-DG-inhibited glucose uptake, lactate production, and ATP production(Fig. 5F–H, all *p* < 0.05), and it suppressed the expression of the HK2, PKM2, GLUT1, and LDHA glycolytic proteins in OC cells (Fig. 6A, all *p* < 0.05). A cell proliferation assay demonstrated the 2-DG glycolysis inhibitor reversed the promoting effect of exosomal TACSTD2 on the proliferation of OC cells (Fig. 6B, *p* < 0.05). Wound-healing assays indicated that the 2-DG glycolysis inhibitor reversed the ability of exosomal TACSTD2 to promote the migration of OC cells (Fig. 6C, *p* < 0.05). Transwell assays indicated that the 2-DG glycolysis inhibitor reversed the ability of exosomal TACSTD2 to promote the migration and invasion of OC cells (Fig. 7A, B, *p* < 0.05). These findings suggested that exosomal TACSTD2 may promote cell proliferation, migration, and invasion in OC cells by mediating glycolysis.

### Exosomal TACSTD2 may promotes progression through the ERBB2/PI3K/AKT/FOXO1 signaling pathway in OC cells

GSEA suggested a potential pattern that there was a positive correlation trend between the high expression of TACSTD2 and the PI3K/AKT pathway. (Fig. 1H), and protein interaction networks predicted that TACSTD2 may regulate the biological behaviour of OC cells via the ERBB2/PI3K/AKT/FOXO1 signaling pathway (Fig. 2A). To investigate the effect of TACSTD2 on related pathway proteins, Western blot analysis was performed. There was significantly lower expression of the ERBB2, p-PI3K, p-AKT, and p-FOXO1 proteins in the SKOV3-shTACSTD2-exo and OVCAR3-shTACSTD2-exo groups than in the SKOV3-exo, SKOV3-shNC-exo, OVCAR3-exo, and OVCAR3-shNC-exo groups (Fig. 7C, all *p* < 0.05). However, no significant difference was observed in the protein expression of PI3K, AKT, or FOXO1. In addition, the SKOV3-TACSTD2-OE-exo and OVCAR3-TACSTD2-OE-exo groups exhibited significantly greater ERBB2, p-PI3K, p-AKT, and p-FOXO1 protein expression compared to the SKOV3-vector-exo and OVCAR3-vector-exo groups (Fig. 7C, all *p* < 0.05), while the expression of PI3K, AKT, and FOXO1 did not significantly differ. Additionally, rescue experiments indicated that overexpression of the p-PI3K, p-AKT, and p-FOXO1 pathway proteins was reversed by treatment with a PI3K inhibitor (LY294002) (Fig. 7D, all *p* < 0.05), with no significant difference observed in the expression of PI3K, AKT, or FOXO1. These findings suggested that exosomal TACSTD2 may regulate cell proliferation, migration, and invasion in OC cells through the ERBB2/PI3K/AKT/FOXO1 pathway.

**Fig. 8:**
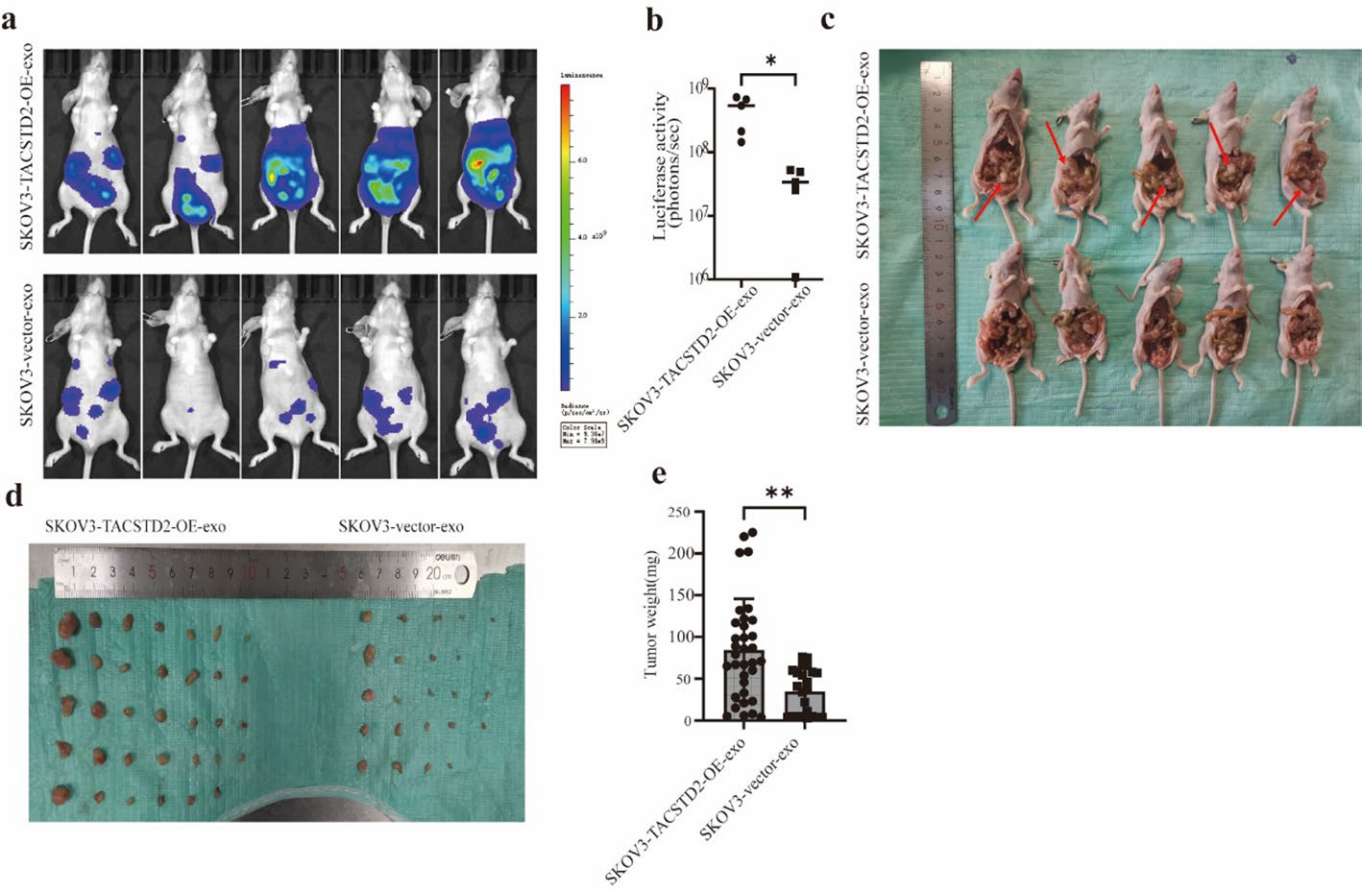
Exosomal TACSTD2 promotes metastasis of ovarian cancer in vivo. **a** Bioluminescence images of abdominal metastasis were detected by an in vivo imaging bioinstrument (*n* = 5). **b** Quantitative radiance values of metastases on abdominal organs. **c** Effects of the exosomes derived from SKOV3-TACSTD2-OE and SKOV3-vector cells on tumour progression in vivo. **d**,** e** The size and weight of the ovarian abdominal implantation tumors from each group. *p < 0.05, **p < 0.01, ***p < 0.001

### Exosomal TACSTD2 promotes OC cell abdominal implantation and metastasis in vivo

To further explore the effect of exosomal TACSTD2 on OC abdominal implantation and metastasis, nude mouse intraperitoneal OC transplantation models were generated using SKOV3-luc + cells. All nude mice were randomly divided into two groups, with five mice in each group. The experimental and control groups were intraperitoneally injected with 50 µg of exosomes from the supernatants of SKOV3-TACSTD2-OE and SKOV3-vector cells every 3 days. The growth and metastasis of OC were detected by a live imaging biological instrument after 8 weeks, and the tumour growth and abdominal implantation were observed by autopsy after euthanasia. The results showed that exosomal TACSTD2 promoted OC abdominal implantation and metastasis in vivo (Fig. 8A–E, all *p* < 0.05).

## Discussion

OC, the deadliest gynaecological malignancy, poses a significant threat to the health and lives of many women, resulting in a substantial emotional and financial burden on patients [32]. Exosomes, which can be readily obtained from various bodily fluids, such as blood, saliva, urine, and ascites, hold promise as valuable biomarkers for liquid biopsies of tumours [33]. These exosomes are enclosed by lipid bilayers, providing protection to their contents during transport and preventing degradation. Given their remarkable stability in body fluids and their pathological characteristics, exosomes play a crucial role in OC, particularly in early diagnosis, prognosis, chemotherapy resistance, and targeted therapy [6, 34, 35]. Further exploration of the relationship between OC and exosomes is essential for understanding the mechanisms driving OC progression and developing novel therapeutic strategies [36].

In this study, OC exosome data from ExoCarta, TCGA, and GEO databases were analyzed to identify DEGs, focusing on TACSTD2. Survival analysis showed a significant association between TACSTD2 expression and OS in OC patients. GSEA revealed potential regulatory mechanisms of TACSTD2, this is an exploratory observation. Although part of our GSEA results did not achieve statistical significance, the observed trend aligns in our dataset, hinting at a potential role that warrants further investigation in larger cohorts. Given its overexpression in most cancer tissues [37], TACSTD2 is an attractive target for cancer therapy. In vivo experiments have demonstrated that TACSTD2 promotes tumour growth and progression, including increased invasiveness and malignancy [38]. Additionally, TACSTD2 serves as a marker of poor prognosis in various solid tumours, such as breast, ovarian, prostate, hepatocellular, and oral squamous cell carcinomas [39–41]. In OC, TACSTD2 is highly expressed in most tissues and is associated with aggressive phenotypes and poor prognosis [42]. This study confirmed TACSTD2 overexpression in OC tissues and cells and verified high levels of exosome-associated TACSTD2 in normal ovarian cell exosomes through Western blot analysis. Although TACSTD2 is known to be highly expressed in OC tissue, the mechanisms underlying its role in OC progression remain unclear [18, 43, 44].

Metastasis is a key stage in cancer progression, often leading to death. In OC, cells primarily metastasize within the peritoneal cavity and greater omentum through passive mechanisms. Exosomes play a crucial role in tumour metastasis [45, 46]. Tumour cell-derived exosomes can be taken up by neighbouring tumour cells, leading to functional changes in the recipient cells [47]. For instance, microRNAs (such as miR-200c/miR-0141) have been shown to promote breast cancer tumour metastasis by activating dormant breast cancer cells [48]. He et al. reported that exosomal miR-21-5p, which is secreted by cancer cells, promotes angiogenesis and vascular permeability by regulating the expression of cyclin D1 (CCND1) in colorectal cancer [46]. In the present study, coculture of exosomal TACSTD2 with OC cells effectively promoted the proliferation, migration, and invasion of OC cells.

Exosomes promote tumour metastasis by enhancing cell invasion, migration, and extracellular matrix reprogramming [49]. Cancer cells face oxidative stress during metastasis, with ROS primarily originating from mitochondrial oxidative metabolism [50]. Cell detachment induces significant oxidative stress, and an excess of reactive oxygen species (ROS) can lead to cell death, specifically apoptosis, which acts as a barrier to metastasis [51]. The primary source of ROS is oxidative metabolism in the mitochondria. The Warburg effect, however, reduces mitochondrial ROS production, increases glycolysis, and enhances nicotinamide adenine dinucleotide phosphate (NADPH) production through the pentose phosphate pathway, which is essential for antioxidant activity [51]. Exosomal miR-122 in breast cancer remodels metabolism in the premetastatic microenvironment, exacerbating metastasis [52]. Additionally, exosomes carrying VEGF stimulate glycolysis in endothelial cells [53], suggesting exosome-mediated metabolic reprogramming is key to metastasis.

Cancer cells reprogram energy metabolism to promote cell proliferation [54]. Aerobic glycolysis, a hallmark of cancer cells, leads to increased glucose consumption and lactic acid production, known as the Warburg effect. This lactic acid alters the microenvironment, promoting cancer cell proliferation and migration [31]. Increased glycolysis in cancer cells is driven by elevated expression of key enzymes like HK2 and PKM2, with HK2 promoting tumor glycolysis and metastasis [55–57]. The present study shows that exosomal TACSTD2 enhances glucose uptake, lactate and ATP production, and the expression of key glycolytic proteins (HK2, PKM2, GLUT1, LDHA) in OC cells. This promotes glycolysis, leading to increased proliferation, migration, and invasion of OC cells.

To elucidate the underlying mechanism by which TACSTD2 promotes metastasis and glycolysis in OC cells, GSEA was performed, and the results suggested a trend that TACSTD2 is associated with the PI3K/AKT pathway. In metastatic urothelial carcinoma, TACSTD2 and ERBB2 are both highly expressed across molecular subtypes, suggesting a potential link between their co-expression [58]. ERBB2 recruitment enhances PI3K/AKT signaling in prostate cancer cells, serving as a key mediator of EGF-induced proliferation [59]. In neuroendocrine prostate cancer, TACSTD2-driven AKT1 and c-Myc activation enhances lineage plasticity and tumor progression [60]. FOXO1, a key downstream target kinase of the PI3K/AKT pathway, is known to be involved in cell proliferation and glycolysis [61]. Thus, further investigations were conducted to determine whether the effects of TACSTD2 on proliferation and glycolysis are mediated through the PI3K/AKT/FOXO1 signaling pathway.

The present study provides the first evidence that exosomal TACSTD2 promotes the proliferation, metastasis, and glycolysis of OC cells. Furthermore, the present findings suggested that exosomal TACSTD2 achieves this effect by enhancing glycolysis. Regarding the underlying mechanisms, the present findings suggest that exosomal TACSTD2 may promote OC proliferation, metastasis, and invasion by regulating glycolysis. Additionally, the in vivo experiments yielded supporting results. However, it is important to acknowledge the limitations of this study. First, a sufficient number of OC ascites samples were not collected for functional validation, and in-depth animal experiments were not performed for validation. Secondly, although our functional data strongly support the phenotypic effect, future omics research will be indispensable for a comprehensive elucidation of the underlying transcriptional networks. In the subsequent research, we will conduct a comprehensive transcriptomics (RNA sequencing) analysis as well as subsequent chromatin immunoprecipitation sequencing (ChIP-seq) analysis.

Recent studies have shed light on the molecular mechanisms of OC metastasis and have made significant progress in exploring the role of exosomes in regulating this process. Exosomes offer new opportunities for the early diagnosis and treatment of OC with targeted drugs. Tumour growth can be promoted through tumour-derived exosomes in a vectorial manner [48]. Consequently, numerous studies have inhibited the release of tumour exosomes using exosome inhibitors, such as GW4869, calpeptin, manunycin A, Y27632, D-pantethine ethylamine, and promethazine [62]. Therapeutic inhibitors combining anticancer drugs and exosomes have also been investigated [63].

OC metastasis is a complex process influenced by various factors, such as stromal remodelling, immunosuppression, angiogenesis, and alterations in the TME. The TME consists of diverse cellular components and substances, and ascites contain various cell types, including cancer cells, cancer-associated fibroblasts (CAFs), and macrophages. The overall impact of ascites on the biological behaviour of tumours is likely to be a combination of these effects, necessitating additional clinical samples and animal studies for further exploration.

## Conclusions

In conclusion, the present study demonstrated that exosomal TACSTD2 plays a significant role in promoting various aspects of OC progression, including cell proliferation, migration, invasion and glycolysis. Additionally, the present findings suggested that exosomal TACSTD2 may contribute to tumour proliferation and metastasis by enhancing glycolysis in OC cells. These findings not only highlight a novel therapeutic target for OC treatment but also provide valuable insights into the underlying mechanisms of OC metastasis.

## Supplementary Information

Supplementary material 1.

Supplementary material 2.

Supplementary material 3.

Supplementary material 4.

Supplementary material 5.

## Data Availability

The datasets generated and analysed during the current study are available in the GEO repository (https://www.ncbi.nlm.nih.gov/geo/)， the TCGA repository (https://portal.gdc.cancer.gov/), and the ExoCarta repository (http://www.exocarta.org/).

## Abbreviations

CAF: Cancer-associated fibroblasts
CCK-8: Cell Counting Kit-8
DEG: Differentially expressed genes
FBS: Fetal bovine serum
GEO: Gene Expression Omnibus
GEPIA: Gene Expression Profiling Interactive Analysis
GSEA: Gene set enrichment analysis
HCC: Hepatocellular carcinoma
HGSOC: High-grade serous ovarian carcinoma
NACT: Neoadjuvant chemotherapy
NADPH: Nicotinamide adenine dinucleotide phosphate
DLS: Dynamic Light Scattering
OC: Ovarian cancer
OS: Overall survival
PBS: Phosphate buffer saline
PVDF: Polyvinylidene fluoride
RIPA: Radio Immunoprecipitation Assay
ROS: Reactive oxygen species
RT-qPCR: Reverse transcription-quantitative polymerase chain reaction
SD: Standard deviation
SDS-PAGE: Sodium dodecyl sulfate-polyacrylamide gel electrophoresis
SPF: Specific pathogen-free
TBS: Tris-buffered saline
TCGA: The Cancer Genome Atlas
TEM: Transmission electron microscopy
TME: Tumour microenvironment
VEGF: Vascular endothelial growth factor
