# Supplementary material for: Exosomal TACSTD2 promotes invasion, metastasis and glycolysis in ovarian cancer
Source: Discov Oncol. 2025 Dec 2;17:23. doi: 10.1007/s12672-025-04041-6 (PMC12775207; doi:10.1007/s12672-025-04041-6)
Supplement: Supplementary file 1 — Supplementary material 1. [file 12672_2025_4041_MOESM1_ESM.docx]

| **Gene symbol** | **Description** | **logFC** | ***P*-value** | **adj.P.Val** |
| --- | --- | --- | --- | --- |
| CLDN3 | claudin 3 | 9.715766 | ＜0.001 | ＜0.001 |
| EPCAM | epithelial cell adhesion molecule | 7.359509 | ＜0.001 | ＜0.001 |
| **CLDN4** | claudin 4 | 7.249853 | ＜0.001 | ＜0.001 |
| **KRT7** | keratin 7, type II | 6.954176 | ＜0.001 | ＜0.001 |
| **TACSTD2** | tumor-associated calcium signal transducer 2 | 6.808473 | ＜0.001 | ＜0.001 |
| HMGA1 | high mobility group AT-hook 1 | 6.395682 | ＜0.001 | ＜0.001 |
| ST14 | suppression of tumorigenicity 14 | 6.373357 | ＜0.001 | ＜0.001 |
| KRT8 | keratin 8, type II | 6.270759 | ＜0.001 | ＜0.001 |
| PRSS8 | protease, serine, 8 | 6.057319 | ＜0.001 | ＜0.001 |
| CP | ceruloplasmin (ferroxidase) | 5.627952 | ＜0.001 | ＜0.001 |

**Table 1 The top 10 upregulated differently expressed exosomal genes in TCGA**
