# Supplementary material for: Exosomal TACSTD2 promotes invasion, metastasis and glycolysis in ovarian cancer
Source: Discov Oncol. 2025 Dec 2;17:23. doi: 10.1007/s12672-025-04041-6 (PMC12775207; doi:10.1007/s12672-025-04041-6)

The heatmap of the differentially expressed genes in OC. The color bar from red to blue denotes high to low gene expression.


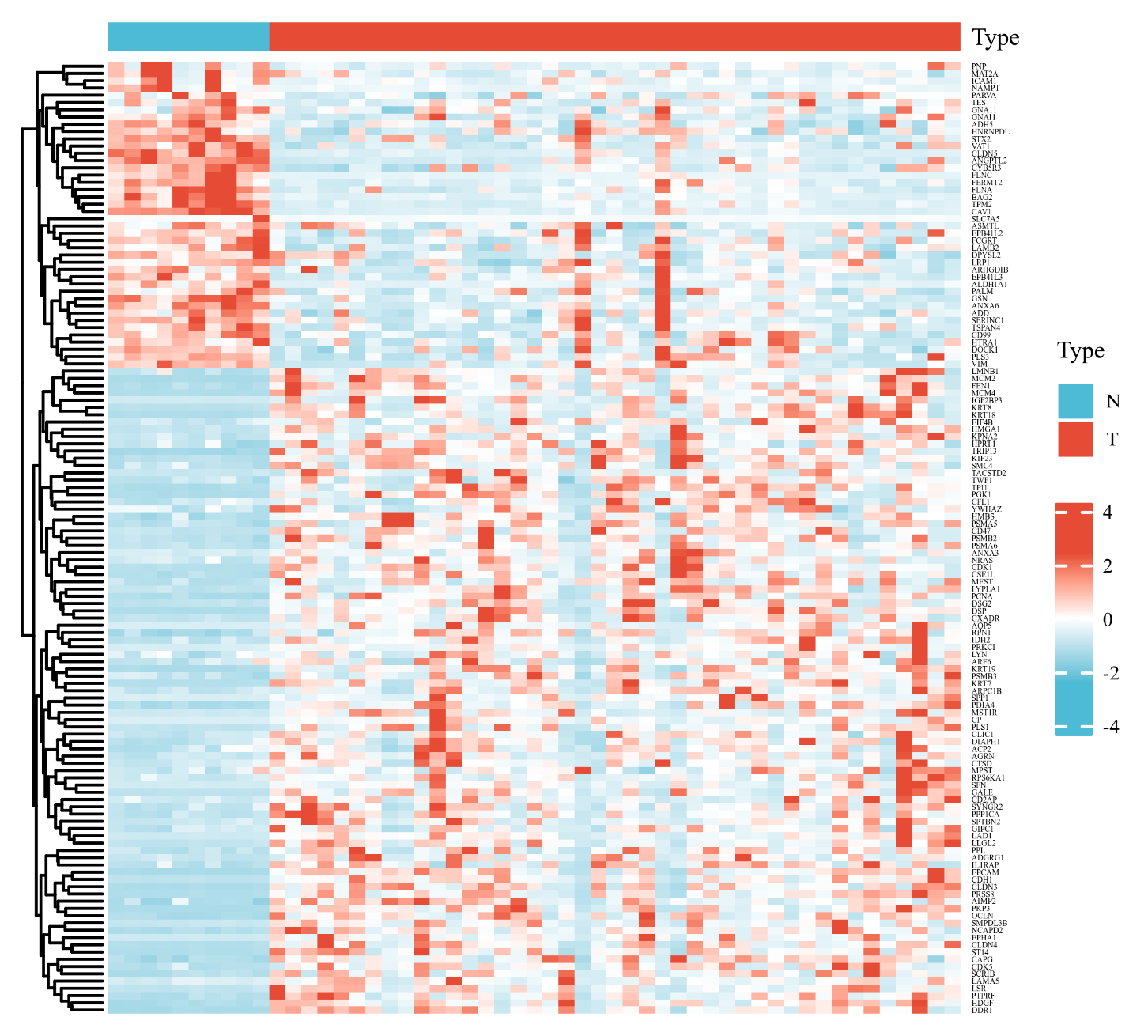

Supplement: Supplementary file 2 — Supplementary material 2. [file 12672_2025_4041_MOESM2_ESM.docx]
